# Relationships between cancer pattern, country income and geographical region in Asia

**DOI:** 10.1186/s12885-015-1615-0

**Published:** 2015-09-03

**Authors:** Chirk Jenn Ng, Chin Hai Teo, Nurdiana Abdullah, Wei Phin Tan, Hui Meng Tan

**Affiliations:** 1 Department of Primary Care Medicine, Faculty of Medicine, University of Malaya, 50603 Kuala Lumpur, Malaysia; 2 Department of Surgery, Thomas Jefferson University Hospital, Philadelphia, PA 19107 USA; 3 Subang Jaya Medical Centre, 47500 Subang Jaya, Malaysia

## Abstract

**Background:**

Cancer incidence and mortality varies across region, sex and country’s economic status. While most studies focused on global trends, this study aimed to describe and analyse cancer incidence and mortality in Asia, focusing on cancer site, sex, region and income status.

**Methods:**

Age-standardised incidence and mortality rates of cancer were extracted from the GLOBOCAN 2012 database. Cancer mortality to incidence ratios (MIRs) were calculated to represent cancer survival. The data were analysed based on the four regions in Asia and income.

**Results:**

Cancer incidence rate is lower in Asia compared to the West but for MIR, it is the reverse. In Asia, the most common cancers in men are lung, stomach, liver, colorectal and oesophageal cancers while the most common cancers in women are breast, lung, cervical, colorectal and stomach cancers. The MIRs are the highest in lung, liver and stomach cancers and the lowest in colorectal, breast and prostate cancers. Eastern and Western Asia have a higher incidence of cancer compared to South-Eastern and South-Central Asia but this pattern is the reverse for MIR. Cancer incidence rate increases with country income particularly in colorectal and breast cancers but the pattern is the opposite for MIR.

**Conclusion:**

This study confirms that there is a wide variation in cancer incidence and mortality across Asia. This study is the first step towards documenting and explaining the changing cancer pattern in Asia in comparison to the rest of the world.

**Electronic supplementary material:**

The online version of this article (doi:10.1186/s12885-015-1615-0) contains supplementary material, which is available to authorized users.

## Background

Recent studies have found that developing countries, particularly those in Asia, are facing a rising cancer incidence. The incidence rates of cancers such as lung and colorectal in some Asian countries have surpassed that of Western countries [1, 2]. This change may be due to the adoption of cancer-related lifestyle such as smoking, alcohol consumption, physical inactivity, obesity and high-fat, low-fibre diet [3]; environmental and occupational risk factors such as air pollution, indoor smoke from household use of solid fuel; and contaminated injections in healthcare settings [4].

Currently, Asia contributes to 48 % of the total number of new cancer cases in the world, of which nearly half are found in China. Similarly, the cancer deaths in Asia constitute 55 % of that in the world. The cancer incidence and mortality in Asia are expected to rise over the next two decades [3]. Asia, comprising mainly developing countries, is also facing a rapid population expansion. This rising cancer incidence would have a significant impact on healthcare burden and individuals’ quality of life across Asia [5].

Previous global studies have found that cancer incidence and mortality vary according to age distribution, sex, location and economic status of a country [1, 2]. Globally, men have been found to have high cancer incidence and mortality rates compared to women [6, 7]. The incidences of cancers in developed countries are higher compared to less developed countries; however, the former have a better survival rate [8]. In addition, many studies have shown wide variations in cancer rates across different parts of the world; some even showing conflicting incidence within the same region [1, 9]. However, most of these studies focused on global trends [1, 9], specific sub-regions of Asia [10] or specific cancer types [11, 12]; there is limited literature on overall cancer epidemiology in Asia.

Therefore, this study, based on the latest GLOBOCAN 2012 data, aimed to describe and analyse the incidence and mortality of cancer in Asia, according to cancer type, sex, region and income status. We believe that this is the first step towards documenting and explaining the changing cancer pattern in Asia in comparison to the rest of the world.

## Methods

We extracted the cancer data for Asia from GLOBOCAN 2012, which provides updated national level cancer statistics for 184 countries in the world [3]. GLOBOCAN uses several estimation methods to produce country-specific incidence and mortality data. For cancer incidence, GLOBOCAN first attempted to obtain the data from the Cancer Incidence in Five Continents (CI5) [13, 14]. If they were not available, national, regional or frequency data were estimated. Similarly, for cancer mortality, GLOBOCAN used national vital registration data from the World Health Organization Statistical Information System where available; otherwise mortality rates were estimated [15]. The data sources and methods for estimating incidence and mortality rates for each country can be found at the GLOBOCAN website [16] and is summarised in Additional file 1. We did not seek ethics approval in this study because we used population-based secondary data from the GLOBOCAN database, which is openly accessible to the public. In addition, the study did not directly involve any individual human subject or identifiable data.

We used age-standardised rates (ASR) to compare the incidence and mortality rates between countries and the ASR were calculated using the World Standard Population and expressed as per 100,000 person-years [17]. Mortality to incidence ratio (MIR) was used as an indirect measure of cancer survival and an MIR of 1.0 indicates no survival [8].

**Fig. 1:**
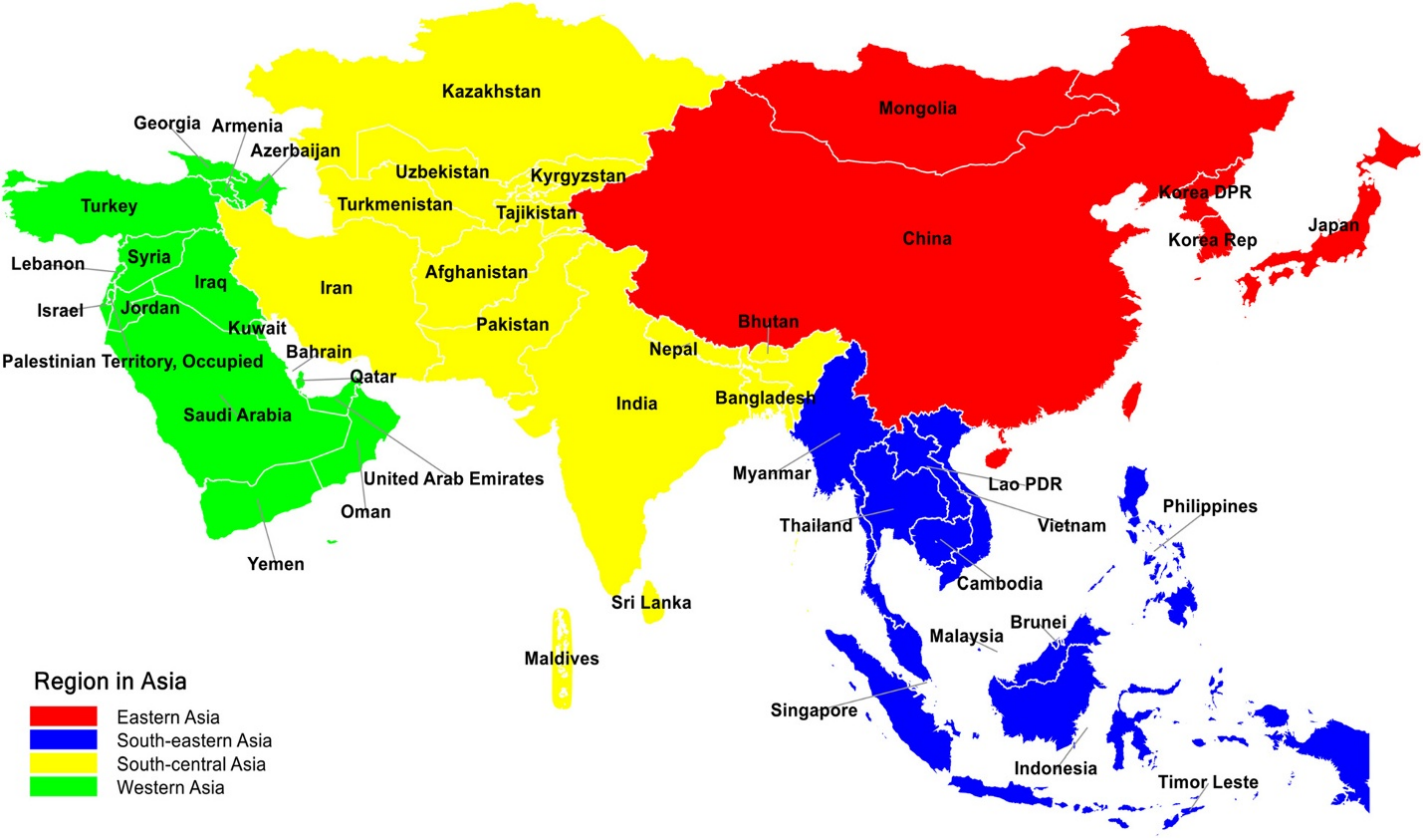
Countries by region in Asia according to United Nation World Population Prospects 2012 [18]

A total of 47 Asian countries were included in this study and they were divided into four subregions according to the United Nation World Population Prospects 2012: Eastern (*n* = 5), South-Eastern (*n* = 11), South-Central (*n* = 14) and Western (*n* = 17) Asia [18] (Fig. 1). We used the gross national income (GNI) per capita for income analysis which is based on the World Bank Atlas methodology (US dollars) [19]. The country income was analysed as a continuous variable and we used linear correlation coefficient to determine the relationships between income and cancer incidence, mortality and MIR.

## Results

### Cancer incidence, mortality and MIR: Asia vs other continents

Asia has a lower overall cancer incidence rate (152.2/100,000; ranked 5/6) compared to more developed continents such as North America (315.6/100,000), Oceania (298.4/100,000) and Europe (255.4/100,000) (Table 1). This pattern is observed in both sexes; however men have a higher incidence of cancer compared to women both in Asia and other continents, except for Africa (women 132.4/100,000 vs men 115.6/100,000).

**Table 1:** Comparison of overall and sex-specific age-standardised incidence, mortality and MIR of all cancers across six continents

All cancers excluding non-melanoma skin cancer	Age-standardized rate (per 100,000 person-years)								
	Overall			Male			Female		
Continent	Inc	Mor	MIR	Inc	Mor	MIR	Inc	Mor	MIR
Northern America	315.6	105.5	0.33	344.2	123.2	0.36	295.4	91.7	0.31
Oceania	298.4	102.5	0.34	338.5	117.8	0.35	264.8	90.0	0.34
Europe	255.4	113.1	0.44	298.9	147.5	0.49	226.7	87.6	0.39
Latin America and Caribbean	177.0	94.9	0.54	187.6	108.4	0.58	170.7	84.7	0.50
Asia	152.2	100.1	0.66	174.1	126.3	0.73	134.3	77.0	0.57
Africa	123.4	89.9	0.73	115.6	92.9	0.80	132.4	88.7	0.67
World	182.3	102.4	0.56	205.4	126.3	0.61	165.3	82.9	0.50

*Inc* Incidence, *Mor* Mortality, *MIR* Mortality to incidence ratio

Similarly, the overall mortality rate in Asia (100.1/100,000; ranked 4/6) was lower than Europe (113.1/100,000), North America (105.5/100,000) and Oceania (102.5/100,000). However, the pattern is the reverse for overall MIR, which is found to be higher in Asia (0.66; ranked 2/6) compared to North America (0.33), Oceania (0.34) and Europe (0.44). This pattern is also observed in both sexes whereby men have a higher MIR compared to women across all continents.

### Cancer incidence and MIR by sex and cancer site in Asia

**Fig. 2:**
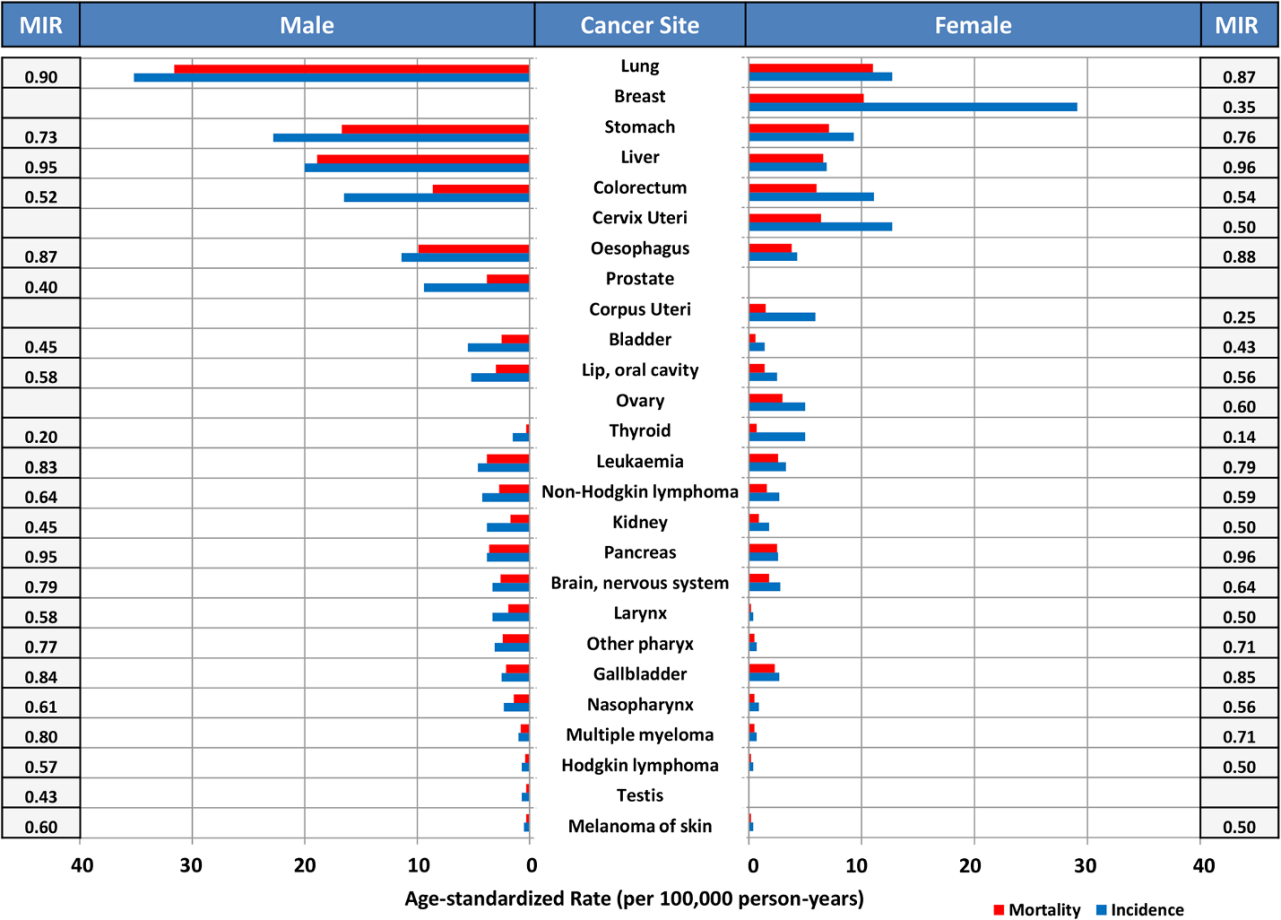
Comparison of age-standardised cancer incidence, mortality and MIR in Asia by sex and cancer site

The most common cancer in men is lung cancer (35.2/100,000), followed by stomach (22.8/100,000), liver (20.0/100,000), colorectal (16.5/100,000) and oesophageal (11.4/100,000) cancers. For women, the most common cancer is breast cancer (29.1/100,000), followed by lung (12.7/100,000), cervical (12.7/100,000), colorectal (11.1/100,000) and stomach (9.3/100,000) cancers (Fig. 2). The cancer incidence is higher in men compared to women in all sites except for gallbladder (male 2.5/100,000; female 2.7/100,000) and thyroid (male 1.5/100,000; female 5.0/100,000) cancers.

For male-specific cancers, prostate cancer is the most common (9.4/100,000) in Asia and it ranked sixth among all the cancers in men. The most common female-specific cancer is breast cancer (29.1/100,000), followed by cervical (12.7/100,000), endometrial (5.9/100,000) and ovarian (5.0/100,000) cancers.

In Asia, the cancer MIRs are the highest in lung (male 0.90; female 0.87), liver (male 0.95; female 0.96) and stomach (male 0.73; female 0.76) cancers. In comparison, colorectal (male 0.52; female 0.54), breast (0.35) and prostate (0.40) cancers have lower MIRs.

### Top five cancer incidence, mortality and MIR in Asia by sex and sub-region

#### All cancers

Overall, the cancer incidence and mortality rates are the highest in East Asia (incidence 186/100,000; mortality 117.7/100,000), followed by West (incidence 168.2/100,000; mortality 103/100,000), South-Eastern (incidence 138.2/100,000; mortality 94.8/100,000) and South-Central (incidence 100.1/100,000; mortality 69.3/100,000) Asia (Table 2). For the MIR, Western Asia (0.61) has the lowest MIR, followed by Eastern (0.63), South-Eastern (0.69) and South-Central (0.69) Asia. In terms of sex, the overall cancer incidence rates are lower in women compared to men in all sub-regions except South-Central Asia. Similarly, the mortality rates and MIRs are observed to be lower in women for all sub-regions.

**Table 2:** Comparison of age-standardised incidence, mortality and MIR of all (excluding non-melanoma skin cancer) and top five cancers in Asia by sub-regions

	Age-standardized rate (per 100,000 person-years)											
Region	Eastern Asia			South-Eastern Asia			South-Central Asia			Western Asia		
Cancer site	Inc	Mor	MIR	Inc	Mor	MIR	Inc	Mor	MIR	Inc	Mor	MIR
Both sexes												
All cancers	186.0	117.7	0.63	138.2	94.8	0.69	100.1	69.3	0.69	168.2	103.0	0.61
Male												
All cancers	225.4	159.3	0.71	147.6	114.1	0.77	98.4	74.8	0.76	192.8	129.3	0.67
Lung	50.4	44.8	0.89	29.6	26.6	0.90	11.9	10.7	0.90	37.6	34.0	0.90
Stomach	35.4	24.0	0.68	8.2	7.3	0.89	9.2	8.5	0.92	11.8	10.2	0.86
Liver	31.9	29.9	0.94	22.2	21.4	0.96	3.7	3.6	0.97	5.0	4.9	0.98
Colorectum	22.4	10.2	0.46	15.2	9.7	0.64	7.0	5.1	0.73	17.6	10.0	0.57
Oesophagus	16.9	14.1	0.83	3.6	3.3	0.92	6.5	6.0	0.92	2.9	2.7	0.93
Female												
All cancers	151.9	80.2	0.53	132.6	79.5	0.60	103.3	64.7	0.63	150.2	81.3	0.54
Breast	27.0	6.1	0.23	34.8	14.1	0.41	28.2	13.5	0.48	42.8	15.1	0.35
Lung	19.2	16.2	0.84	10.5	9.4	0.90	3.4	3.1	0.91	7.1	6.2	0.87
Cervix	7.9	3.3	0.42	16.3	7.9	0.48	19.3	10.9	0.56	4.4	1.9	0.43
Colorectum	14.6	6.8	0.47	10.2	6.4	0.63	5.2	3.8	0.73	12.4	7.1	0.57
Stomach	13.8	9.8	0.71	4.1	3.5	0.85	4.2	3.9	0.93	7.3	6.2	0.85

*Inc* Incidence, *Mor* Mortality, *MIR* Mortality to incidence ratio

#### Lung cancer

Lung cancer has the highest incidence and mortality rates among men for all sub-regions. For both sexes, Eastern Asia has the highest incidence rate (male 50.4/100,000; female 19.2/100,000) whereas South-Central Asia has the lowest incidence rate (male 11.9/100,000; female 3.4/100,000). However, the pattern is the reverse for MIR where Eastern Asia has the lowest MIR (0.84) whereas South-Central Asia has the highest MIR (0.91).

#### Breast cancer

The incidence of breast cancer is the highest in Western Asia (42.8/100,000), followed by South-Eastern (34.8/100,000), South-Central (28.2/100,000) and Eastern (27.0/100,000) Asia. Conversely, breast cancer MIR is the lowest in Eastern Asia (0.23) and highest in South-Central Asia (0.48).

#### Cervical cancer

South-Central Asia has the highest cervical cancer incidence rate (19.3/100,000), followed by South-Eastern (16.3/100,000), Eastern (7.9/100,000) and Western (4.4/100,000) Asia. South-Central Asia has the highest MIR (0.56) while Eastern Asia has the lowest MIR (0.42) for cervical cancer.

#### Colorectal cancer

Eastern Asia has the highest incidence of colorectal cancer (male 22.4/100,000; female 14.6/100,000) with the lowest MIR (male 0.46; female 0.47) for both men and women in Asia. Conversely, the lowest incidence of colorectal cancer is found in South-Central Asia (male 7.0/100,000; female 5.2/100,000), which also has the highest MIR (male 0.73; female 0.73).

#### Liver cancer

Liver cancer is one of the top five cancers among Asian men. It is particularly common in Eastern (31.9/100,000) and South-Eastern (22.2/100,000) Asia compared to South-Central (3.7/100,000) and Western (5.0./100,00) Asia. The MIRs are high in all sub-regions of Asia (range 0.94–0.98).

#### Stomach cancer

Eastern Asia has the highest incidence of stomach cancer for both men (35.4/100,000) and women (13.8/100,000) while the incidence is the lowest in South-Eastern (male 8.2/100,000; female 4.1/100,000) and South-Central (male 9.2/100,000; female 4.2/100,000) Asia. However, the MIR is the lowest in Eastern Asia (male 0.68; female 0.71) compared to South-Central Asia, which has the highest MIR (male 0.92; female 0.93) for stomach cancer.

#### Oesophageal cancer

Oesophageal cancer is also common among Asian men with the incidence being the highest in Eastern Asia (16.9/100,000) and it is less common in other sub-regions of Asia (Western 2.9/100,000; South-Eastern 3.3/100,000; South-Central 6.5/100,000). Although, Eastern Asia has the highest incidence of oesophageal cancer, it has the lowest MIR (0.83) in Asia.

### Top five cancer incidence, mortality and MIR in Asia by sex and country income group

#### Top five cancers in men

**Fig. 3:**
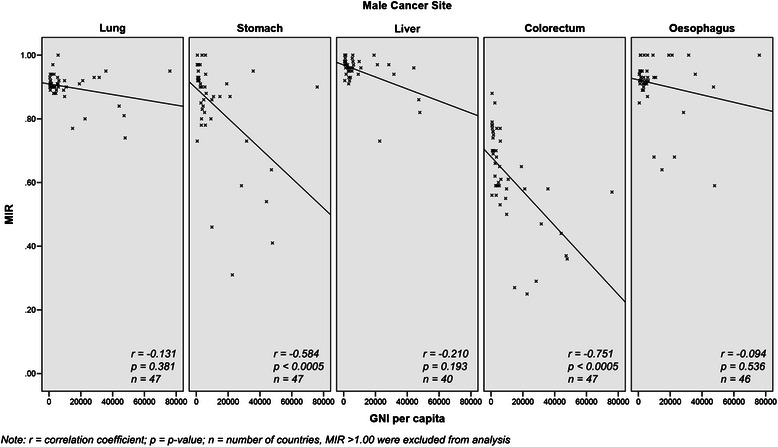
Comparison of top five male cancers MIR to GNI per capita in Asian countries

In Asia, the incidence of colorectal cancer increases with country income (*r* = 0.519, *p* < 0.0005) but the pattern is the opposite for oesophageal cancer (*r* = −0.369, *p* = 0.011) in men (Table 3); however, this is not observed in lung, liver and stomach cancers. For mortality, there is a positive correlation between colorectal cancer and country income group (*r* = 0.307, *p* = 0.036) and the reverse pattern is observed in oesophageal cancer (*r* = −0.388, *p* = 0.007). The MIR is lower in higher income countries for stomach (*r* = −0.584, *p* < 0.0005) and colorectal (*r* = −0.751, *p* < 0.0005) cancers (Fig. 3). This pattern is not observed in lung, oesophageal and liver cancers.

**Table 3:** Correlation between GNI per capita and incidence and mortality of all and top five cancers in men and woman in Asia

Male					Female				
Cancer site	Correlation coefficient				Cancer site	Correlation coefficient			
	Inc	*p*	Mor	*p*		Inc	*p*	Mor	*p*
All cancers	0.240	0.104	−0.225	0.129	All cancers	0.177	0.234	−0.163	0.272
Lung	0.173	0.246	0.152	0.307	Breast	0.534	<0.0005	0.013	0.928
Stomach	−0.072	0.631	−0.207	0.162	Lung	0.142	0.340	0.122	0.415
Liver	−0.060	0.689	−0.068	0.648	Cervix	−0.354	0.015	−0.528	<0.0005
Colorectum	0.519	<0.0005	0.307	0.036	Colorectum	0.604	<0.0005	0.463	0.001
Oesophagus	−0.369	0.011	−0.388	0.007	Stomach	0.030	0.840	−0.142	0.340

*Inc* Incidence, *Mor* Mortality, *p p*-value

#### Top five cancers in women

**Fig. 4:**
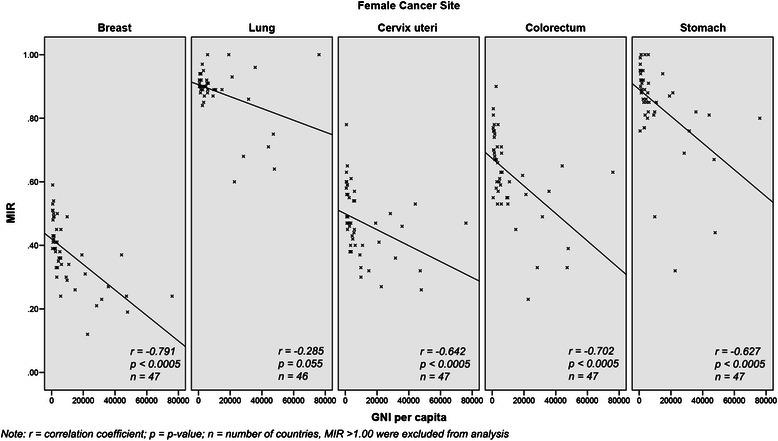
Comparison of top five female cancers MIR to GNI per capita in Asian countries

For Asian women, the incidence of breast (*r* = 0.534, *p* < 0.0005) and colorectal (*r* = 0.604, *p* < 0.0005) cancer increases with country income (Table 3), while the reverse is true for cervical cancer (*r* = 0.354, *p* = 0.015). For mortality, it is higher in women with cervical cancer from lower income countries (*r* = −0.528, *p* < 0.0005) but the reverse pattern is observed in colorectal cancer (*r* = 0.463, *p* = 0.001). There is an inverse correlation between MIR and income group for breast (*r* = −0.791, *p* < 0.0005), cervical (*r* = −0.642, *p* < 0.0005), colorectal (*r* = −0.702, *p* < 0.0005), and stomach (*r* = −0.627, *p* < 0.0005) cancers (Fig. 4).

## Discussion

This study highlights a few interesting patterns of cancer incidence and MIR in Asia: (1) Asia has one of the lowest incidence of overall cancer rates but it has the second highest MIR in the world; (2) There are similarities in the leading cancers diagnosed in Asia and those in more developed continents such as Australia, Europe and Northern America, apart from prostate, cervical and liver cancers. For men, prostate cancer is the most common cancer in the developed countries while liver cancer is relatively more common in Asia. For women, cervical and liver cancer are more common in Asia compared to the developed countries; (3) there is a variation in the cancer incidence and MIR across different sub-regions of Asia, with Eastern and Western Asia having a higher cancer incidence and lower MIR compared to South-Eastern and South-Central Asia.

Globally, the incidence of and mortality attributed to cancer are rising which is most likely due to ageing population as well as reductions in childhood mortality and deaths related to infectious diseases, such as respiratory and gastrointestinal infections [20–23]. However, there are significant differences in cancer burden across the world and they are mainly attributed to the variation in the age structure; prevalence of risk factors such as nutrition and diet, infection and tobacco; environmental and occupational factors; availability and use of cancer screening services; and access to and quality of the cancer treatment [4, 9, 24–26]. Asia, which comprises mostly developing countries, has been having a steady rise in the incidence of cancer over the years [9]. Breast, lung, stomach, colorectal and liver cancers constitute the most common cancers in Asia.

Prostate cancer is the most common male-specific cancer in Asia and it ranks sixth among all cancers in Asian men, compared to developed countries where prostate cancer is the most common cancer among men [1, 3]. It has been well established that Asian particularly Chinese men have a lower risk of prostate cancer compared to black and white men [27]. In fact, recently, there is an observed rise in the incidence of prostate cancer in countries such as the Philippines, Japan, China and Singapore and this is most likely due to the increased uptake of prostate specific antigen (PSA) screening [1, 3, 28]. The most common female-specific cancer in Asia is breast cancer, which is also the most common cancer among Asian women; this is followed by cervical, endometrial and ovarian cancer. This pattern is similar to those of the developed countries except for endometrial cancer which is more common than cervical cancer in the developed countries [1, 3].

Nasopharyngeal cancer are more commonly seen in the Asian population compared to those in other continents. South-Eastern Asia countries, such as Malaysia, Vietnam, Singapore and Indonesia, contribute to most of the nasopharyngeal cancer cases, which are associated with Epstein-Barr virus, consumption of preserved food, tobacco use and genetic factors [29]. The incidence rate for lip and oral cancers is also high in South Asian countries such as Sri Lanka, Pakistan, Bangladesh and India, contributing to most of the cases in Asia [3]. Aetiological agents for lip and oral cancers such as betel quid and areca nut chewing are commonly practised by the South Asians. Other risk factors include smoking, alcohol use, human papillomavirus (HPV) infection, obesity and genetic factors [30].

In this study, one striking finding is the high cancer incidence in Eastern and Western Asia and this is observed in both sex- and non-sex specific cancers (except cervical cancer). It is likely that lifestyle differences between these sub-regions contribute to the variation in cancer incidence. For instance, the prevalence of cigarette smoking among men is much higher in China (57.4 %) compared to India (32.7 %) and Pakistan (27.3 %) [31]. This can explain the relative higher incidence of lung and oesophageal cancer in Eastern Asia. Increasingly, the developed and developing countries in Asia are adopting a more Westernised lifestyle, which include a high fat, low fibre diet [32]. Countries in East Asia, such as Japan and South Korea, see a rise in the incidence of colorectal cancer, which is associated with a low-fibre diet [8]. A high-fat, high calorie diet and sedentary lifestyle in some Asian countries also contributed to the rising incidence of obesity, which is associated with breast cancer [33, 34]. In some Asian population, such as the Chinese, weight gain is related to an increased risk of postmenopausal breast cancer even in relatively thin women [35]. This may explain the observed high incidence of breast cancer in Western Asia, which has among the highest prevalence of female obesity in Asia [36]. Environmental factors such as air pollution may also play an important role as the developing countries become more industrialised [4].

The high incidence of oesophageal cancer, particularly squamous cell carcinoma, is likely to be associated with higher alcohol consumption [37], which is seen in some Eastern Asian countries [38, 39]. For example, between 2008 and 2010, the total adult per capita consumption of pure alcohol in Japan was 7.2 l (male 10.4 l vs female 4.2 l) and in China was 6.7 l (male 10.9 l vs female 2.2 l) compared to less than 1.0 l in Saudi Arabia, Iran, Pakistan, Indonesia and Myanmar [40]. However, these data reflect associations rather than causality and does not take into consideration the lag time between alcohol consumption and the development of oesophageal cancer.

Another important observation is the high incidence of infection-related cancers, such as liver, stomach and cervical cancers, in Eastern Asia. For example, 75–80 % of liver cancer in Eastern and South-Eastern Asia are associated with chronic hepatitis B virus infection [41]. This is in contrast to the West, where alcohol consumption is the main aetiological factor for liver cancer [37, 42]. In Japan and Korea, where the incidence of stomach cancer is one of the highest in Asia, the prevalence of *H. Pylori* infection among asymptomatic adults aged more than 40 years is as high as 80–90 % [43].

In Asia, South-Central Asia has the lowest cancer incidence in both men and women for all cancers except cervical cancer. This is despite the relatively low incidence of reported HPV infection in South Asian countries, such as India [44]. Possible explanation for this high incidence of cervical cancer include sexual practices and malnutrition in this subregion [45, 46].

In Asia, the MIRs as well as absolute mortality rates are particularly high in lung, liver and stomach cancers in both men and women. Patients with these cancers often present late as there is no effective screening method [47]. In comparison, colorectal, breast, cervical and prostate cancers have lower MIRs and this is because they have a longer latency period and effective screening methods. However, despite this, the MIRs for breast (0.35), prostate (0.4), cervical (0.5) and colorectal (male 0.52 vs female 0.54) cancers are high, compared to those of the developed countries [8]. Studies have found that in Asia, postmenopausal women with breast cancer are more likely to be oestrogen-receptor negative, which carry a poor prognosis, compared to those in North America [8]. In addition, population-based breast cancer screening programme is only available in few countries in Asia, such as Japan, Singapore, South Korea and Taiwan [48]. For cervical cancer, it is worrying to see that the both the incidence and MIR were high in South-Central Asia, of which India accounted for 27 % of the total cervical cancer deaths in the world [1]. The high mortality of cervical cancer in this subregion may be attributed to unavailability of population screening and HPV vaccine. For example, in India, Nepal and Bangladesh, the proportion of eligible women who underwent a pelvic examination and pap smear in the past 3 years was less than 10 % compared to 44 % in Israel [49]. In prostate cancer, it was reported that the uptake for prostate cancer screening in Japan remains low; the delay in help seeking in men and, therefore, late diagnosis may explain the higher than expected MIR [50]. For colorectal cancer, most Asian countries do not have a structured mass screening programme except Taiwan and South Korea. Colorectal cancer screening is provided freely in Taiwan and subsidised in South Korea [51].

There are several limitations in this study. There is a wide variation in data quality and accuracy from GLOBOCAN. Only a small number of Asian countries have data on cancer incidence rates in the CI5 database, and only a few have complete national death data. In addition, we also excluded several countries from the correlation analysis of income vs MIR because the reported MIR was more than 1.0.

## Conclusions

This study confirms that there is a wide variation in cancer incidence and mortality across Asia. This raises the concern that the rapidly developing and westernised Asia may be facing a ‘cancer epidemic’ in the near future. While the more developed and higher income countries in Eastern and Western Asia still register a higher cancer incidence than the less developed and lower income South-Central and some South-Eastern Asia countries, the latter struggle to cope as evidenced by the high MIR. This is particularly worrying as lifestyle- and infection-related aetiological factors remain uncurbed in these resource-poor countries.

This calls for an urgent investigation into the causes of high cancer MIR in South-eastern and South-central subregions as well as low income countries in Asia so that effective interventions can be developed to improve cancer survival. More systematic and accurate collection of epidemiological data are necessary to monitor the trend and evaluate the effectiveness of these interventions. This is a task that requires collaborations between local government and local, regional and international non-governmental organisation, such as WHO. There should be a platform for countries within and outside Asia to share and brainstorm strategies to improve cancer detection and survival so as to reduce the burden of cancer on individuals, family and the nation.

## Additional file

Additional file 1: **Data sources and methods for estimating incidence and mortality rates for each Asian country.** (DOCX 26 kb)

## Abbreviations

MIR: Mortality to incidence ratio
CI5: Cancer incidence in five continents
ASR: Age-standardised rates
GNI: Gross national income
PSA: Prostate specific antigen
HPV: Human papillomavirus
Inc: Incidence
Mor: Mortality
