# Supplementary material for: Relationships between cancer pattern, country income and geographical region in Asia
Source: BMC Cancer. 2015 Sep 3;15:613. doi: 10.1186/s12885-015-1615-0 (PMC4558762; doi:10.1186/s12885-015-1615-0)
Supplement: Additional file 1: — Data sources and methods for estimating incidence and mortality rates for each Asian country. (DOCX 26 kb) [file 12885_2015_1615_MOESM1_ESM.docx]

**Additional file 1 – Data sources and methods for estimating incidence and mortality rates for each country in Asia.**

| **COUNTRY** | **Data source** | | **Methods** | | **COUNTRY** | **Data source** | | **Methods** | |
| --- | --- | --- | --- | --- | --- | --- | --- | --- | --- |
|  | **^#^Inc** | **^$^Mor** | **^Inc** | ***Mor** |  | **^#^Inc** | **^$^Mor** | **^Inc** | ***Mor** |
| Afghanistan | G | 6 | 9 | 5 | Malaysia | C | 2 | 6 | 5 |
| Armenia | G | 3 | 5 | 2 | Maldives | G | 6 | 9 | 5 |
| Azerbaijan | G | 2 | 5 | 2 | Mongolia | D | 5 | 2 | 2 |
| Bahrain | A | 3 | 1 | 1 | Myanmar | G | 6 | 9 | 5 |
| Bangladesh | F | 6 | 8 | 5 | Nepal | G | 6 | 9 | 5 |
| Bhutan | D | 6 | 2 | 5 | Oman | A | 3 | 2 | 5 |
| Brunei | F | 5 | 5 | 2 | Pakistan | E | 6 | 6 | 5 |
| Cambodia | G | 6 | 9 | 5 | Philippines | B | 2 | 6 | 2 |
| China | C | 4 | 3 | 1 | Qatar | A | 3 | 2 | 5 |
| Georgia | G | 2 | 5 | 2 | Saudi Arabia | D | 6 | 1 | 5 |
| India | C | 5 | 6 | 4 | Singapore | A | 1 | 1 | 1 |
| Indonesia | F | 6 | 6 | 5 | Sri Lanka | D | 6 | 2 | 5 |
| Iran, IR | C | 6 | 6 | 5 | State of Palestine | F | 6 | 8 | 5 |
| Iraq | F | 6 | 8 | 5 | Syrian AR | G | 6 | 9 | 5 |
| Israel | A | 2 | 1 | 1 | Tajikistan | G | 3 | 5 | 2 |
| Japan | B | 1 | 3 | 1 | Thailand | B | 3 | 6 | 4 |
| Jordan | D | 5 | 1 | 5 | Timor-Leste | G | 6 | 9 | 5 |
| Kazakhstan | G | 2 | 5 | 2 | Turkey | C | 6 | 6 | 5 |
| Korea, DR | G | 6 | 9 | 6 | Turkmenistan | G | 2 | 5 | 1 |
| Korea, R | A | 2 | 1 | 1 | United Arab Emirates | D | 6 | 2 | 5 |
| Kuwait | A | 2 | 1 | 1 | Uzbekistan | G | 2 | 5 | 2 |
| Kyrgyzstan | G | 2 | 5 | 1 | Viet Nam | E | 4 | 5 | 3 |
| Lao PDR | G | 6 | 9 | 5 | Yemen | E | 6 | 7 | 5 |
| Lebanon | D | 6 | 2 | 5 |  |  |  |  |  |

**Legends:**

**Inc**: Incidence

**Mor**: Mortality

**^#^ A**: National data or high quality regional (coverage greater than 50%); **B**: Regional (coverage between 10% and 50%); **C**: Regional (coverage lower than 10%); **D**: National data (rates); **E**: Regional data (rates); F:Frequency data; **G**: No data.

**^$^ 1**: High quality (CI5) complete vital registration; **2**: Medium quality (CI5) complete vital registration; **3**: Low quality (CI5) complete vital registration; **4**: Incomplete or sample vital registration; **5**: Other sources (cancer registries, verbal autopsy surveys etc.); **6**: No data.

**^** **1**: Rates projected to 2012; **2**: Most recent rates applied to 2012 population; **3**: Estimated from national mortality by modelling, using incidence mortality ratios derived from recorded data in country-specific cancer registries; **4**: Estimated from national mortality estimates by modelling, using incidence mortality ratios derived from recorded data in local cancer registries in neighbouring countries; **5**: Estimated from national mortality estimates using modelled survival; **6**: Estimated as the weighted average of the local rates; **7**: One cancer registry covering part of a country is used as representative of the country profile; **8**: Age/sex specific rates for "all cancers" were partitioned using data on relative frequency of different cancers (by age and sex); **9**: The rates are those of neighbouring countries or registries in the same area.

***** **1**: Rates projected to 2012; **2**: Most recent rates applied to 2012 population; **3**: Estimated as the weighted average of regional rates; **4**: Estimated from national incidence estimates by modelling, using country-specific survival; **5**: Estimated from national incidence estimates using modelled survival; **6**: The rates are those of neighbouring countries or registries in the same area.

**Summary of data quality and estimation method used for Asian countries.**

| **Quality and Accuracy** | **Data Quality** | | | | **Estimation Method** | | | |
| --- | --- | --- | --- | --- | --- | --- | --- | --- |
|  | **Inc** | **Count (%)** | **Mor** | **Count (%)** | **Inc** | **Count (%)** | **Mor** | **Count (%)** |
| Highest | A | 7 (14.9) | 1 | 2 (4.3) | 1 | 7 (14.9) | 1 | 9 (19.1) |
|  | B | 3 (6.4) | 2 | 11 (23.4) | 2 | 7 (14.9) | 2 | 9 (19.1) |
|  | C | 5 (10.6) | 3 | 6 (12.8) | 3 | 2 (4.3) | 3 | 1 (2.1) |
|  | D | 6 (12.8) | 4 | 2 (4.3) | 4 | 0 (0.0) | 4 | 2 (4.3) |
|  | E | 3 (6.4) | 5 | 4 (8.5) | 5 | 10 (21.3) | 5 | 25 (53.2) |
|  | F | 4 (8.5) | 6 | 22 (46.8) | 6 | 8 (17.0) | 6 | 1 (2.1) |
|  | G | 19 (40.4) |  |  | 7 | 1 (2.1) |  |  |
|  |  |  |  |  | 8 | 3 (6.4) |  |  |
| Lowest |  |  |  |  | 9 | 9 (19.1) |  |  |
| TOTAL |  | 47 (100.0) |  | 47 (100.0) |  | 47 (100.0) |  | 47 (100.0) |
